# DNA Methylation Aberrant in Atherosclerosis

**DOI:** 10.3389/fphar.2022.815977

**Published:** 2022-03-03

**Authors:** Yao Dai, Danian Chen, Tingting Xu

**Affiliations:** Department of Cardiology, The First Affiliated Hospital of Anhui Medical University, Hefei, China

**Keywords:** atherosclerosis, DNA methylation, hyperhomocysteinemia, oxidative stress, aging

## Abstract

Atherosclerosis (AS) is a pathological process involving lipid oxidation, immune system activation, and endothelial dysfunction. The activated immune system could lead to inflammation and oxidative stress. Risk factors like aging and hyperhomocysteinemia also promote the progression of AS. Epigenetic modifications, including DNA methylation, histone modification, and non-coding RNA, are involved in the modulation of genes between the environment and AS formation. DNA methylation is one of the most important epigenetic mechanisms in the pathogenesis of AS. However, the relationship between the progression of AS and DNA methylation is not completely understood. This review will discuss the abnormal changes of DNA methylation in AS, including genome-wide hypermethylation dominating in AS with an increase of age, hypermethylation links with methyl supply and generating hyperhomocysteinemia, and the influence of oxidative stress with the demethylation process by interfering with the hydroxyl-methylation of TET proteins. The review will also summarize the current status of epigenetic treatment, which may provide new direction and potential therapeutic targets for AS.

## Introduction

Atherosclerosis (AS) is the pathological basis in heavy cardiovascular diseases like unstable angina pectoris, acute myocardial infarction, and abdominal aortic aneurysm (Tabas et al., 2015). Due to hidden onset, these diseases are difficult to detect and diagnose in the early stage, and serious damage has been caused and a heavy economic burden is imposed once discovered (Zhao et al., 2019). AS is a complex pathological processes beginning with the accumulation of lipids in damaged vessel walls and oxidative modification to oxidized low-density lipoproteins (ox-LDLs), activation immune system, rapid reaction inflammatory, followed with endothelial cell (ECs) activation, arterial smooth muscle cell (SMC) proliferation, activation of macrophages, and formation of foam cells (Tabaei and Tabaee, 2019). In this process, monocytes/macrophages, vascular smooth muscle cells (VSMCs), and vascular endothelial cells (VECs) are all involved in the pathological process (Yuan et al., 2020; Botts et al., 2021). In clinical practice, AS is associated with certain diseases like hyperlipidemia, diabetes mellitus, hyperhomocysteinemia, and hyperuricemia (Jiang et al., 2021). On the other hand, male, smoking history, obesity, aging, and poor lifestyle can also lead to AS (Rizzacasa et al., 2019).

The epigenetic modifications mostly include DNA methylation, histone modification, and non-coding RNAs. Nowadays, genotyping methods supported by statistical and computational approaches enabled large-scale genome-wide association studies (GWAS), in which a large number of genetic variants are investigated in a search for links with the trait of interest. Genetic variants that are too rare to be detected by GWAS are aggregated into subsets, and their frequency is compared between patients and control. More recently, next-generation sequencing (NGS) technologies have also better improved this problem (Veljkovic et al., 2018). Epigenetic modifications have been proved in so many medical research, such as breast cancer, lung cancer, and thyroid cancer, and already contributed major new molecular biology markers, genes, or pathways (Patani et al., 2020). Among these, DNA methylation is the most well-studied epigenetic mark partly due to the development of multiple approaches to assay it, including microarrays and bisulfite sequencing, and its relative stability allowing for profiling of previously collected stored DNA samples (Rask-Andersen et al., 2016). In recent years, GWAS of coronary heart disease (CHD) have identified some genetic risk factors. On the other hand, the association between phenotype and DNA methylation changes across the genome is assessed through epigenome-wide association studies (EWASs) (Xia et al., 2021). So, in this article, we want to illustrate the specific regulatory mechanisms of DNA methylation in the pathogenesis of AS. The main objectives of this review are (1) to describe the dynamic balance and adjustment process of methylation and demethylation, (2) to study the DNA methylation changes in lipid oxidation, vascular smooth muscle cells, vascular endothelial cells, mononuclear-macrophage activation, oxidative stress, and vascular aging, (3) to analyze the influence of linked risk factors like homocysteine, aging, and metabolism on DNA methylation in AS, and (4) to explore current clinical methylation studies on therapy in AS patients.

### DNA Methylation/Demethylation

DNA methylation (DNAme) is one of the most well-understood epigenetic modifications concentrating to happen in the CPG islands region (CGIs) (Papin et al., 2020). The presence of DNAme in promoter and enhancer will be associated with gene silencing (Stratton et al., 2019). The level of methylation is inversely proportional to the level of gene expression, and the position of methylation in a transcription unit affects its relationship to gene control.

Nowadays, accumulating evidence has suggested that DNAme may be reversible in mammalian cells. The regulation by DNAme is at times quite dynamic (Parry et al., 2021). Abnormal increases or decreases in DNA methylation contribute to or are closely related to different diseases like cancers and atherosclerosis (Ehrlich, 2019). The DNAme turnover depends on the DNA methyltransferases (DNMTs) writing the methylation mode, and ten–eleven translocation (TET) enzymes remove their activity (Parry et al., 2021).

**FIGURE 1:**
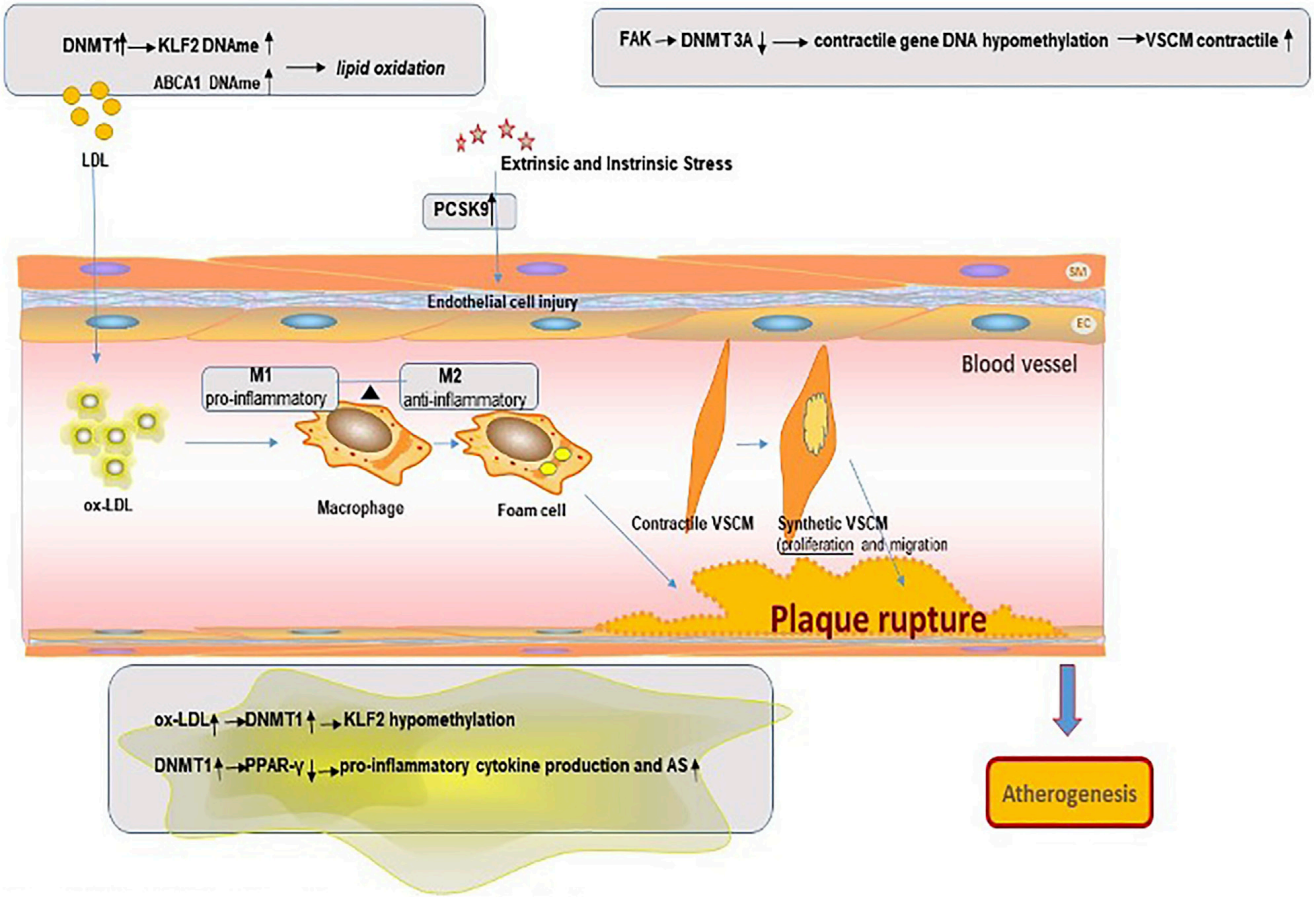
S-Adenosylmethionine is provided by one-carbon metabolism and generates homocysteine.

DNAme is an epigenetic modification catalyzed by DNA methyltransferases (DNMTs). Adding methyl (CH_3_) from S-adenosylmethionine (SAM) to the C5 position of the cytosine base will convert cytosine–guanosine into CpGs (Schiano et al., 2020). SAM is provided by one carbon metabolism and generates homocysteine (HCys) (Shen et al., 2020) (Figure 1)

DNMTs family include DNMT1, 2, 3A, 3B, and 3L. DNMT1 is the key enzyme to maintain DNAme, which is responsible for maintaining the existing methylation patterns (Gujar et al., 2019). DNMT2 lacks DNA methyltransferase activity, mainly catalyzing the methylation of RNA as a transfer RNA (tRNA) methyltransferase. DNMT3A and DNMT3B mediate *de novo* methylation in undifferentiated cells, which contribute to the formation and subsequent maintenance of DNAme marks. DNMT3L mainly regulates DNAme during early embryogenesis, actually expressed only in germ cells and embryonic stem cells but not in somatic cells (Veland et al., 2019).

**FIGURE 2:**
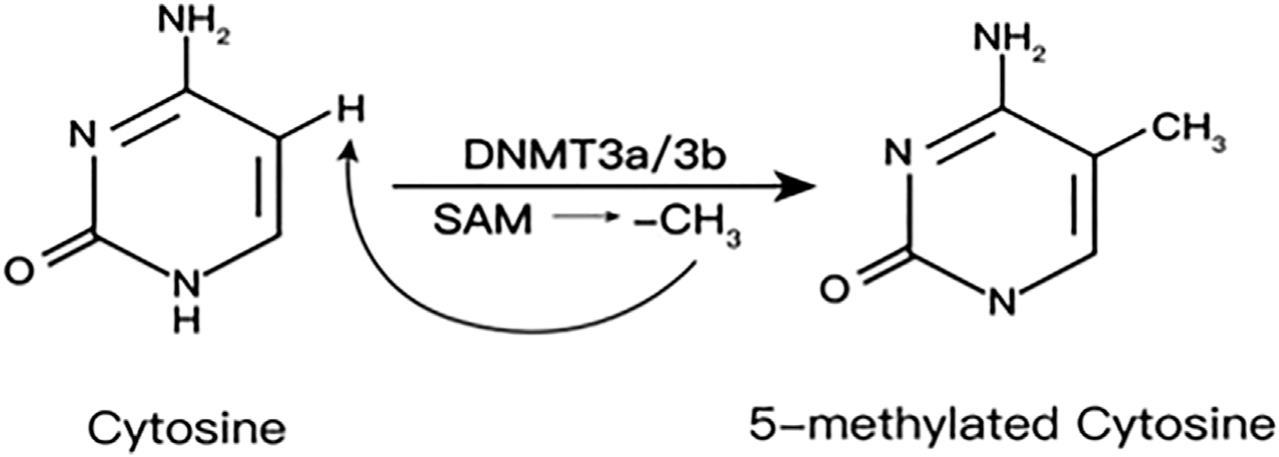
Mechanism of DNA methylation in the pathogenesis of atherosclerosis plaque.

In the process of catalytic methylation, the various enzymes interact with each other. Research shows that knockout Dnmt3a and Dnmt3b in mouse embryonic cells will result in a gradual loss of DNAme over time, indicating that the involvement of Dnmt3a/Dnmt3b also plays an important role in maintaining DNAme profiles during embryonic development (Chen et al., 2003) (Figure 2).

In recent years, the discovery of 5-hydroxymethyl cytosine (5-hmC) has opened a new horizon of the process in removing methyl marks from DNA (Bhutani et al., 2011). The TET family, including TET1, 2, and 3, catalyzes the oxidation of 5-mC to 5-hmC to get the methyl marks removed.

TET2 dominates a protective role in preventing AS by repressing the VSMC phenotype transformation, protecting ECs from damage and dysfunction and inhibiting inflammation (Liu et al., 2018). All of the TETs are Fe(II)-dependent dioxygenases and require Fe (II) as cofactors. Oxidative stress will block the reduction of Fe(III) back to Fe(II), causing less regeneration of active enzyme (Niu et al., 2015).

### DNA Methylation Abnormalities in AS

Multiple studies have shown that DNA methylation is associated with atherosclerotic phenotypes. Aberrant DNA methylation, including hypermethylation and hypomethylation, plays an important role in AS (Chistiakov et al., 2017). In healthy individuals, CGIs in the promoter region of genes are, in general, hypomethylated, whereas CpGs in the non-promoter region are hypermethylated (Khyzha et al., 2017). Global DNA hypomethylation (which is known as DNA hypomethylation of non-promoter regions) can cause structural changes and the instability of chromosomes because of the initiation of transcription at incorrect regions and the high transcriptional activity in sites that are usually silent (Zhang et al., 2021). Genome-wide DNA hypomethylation leads to the expression of potentially harmful genes and also the high expression of genes that are meant to be silent. Conversely, genome-wide DNA hypermethylation causes the inactivation of disease-suppressor genes or protective genes, gene mutation, and allelic loss.

In total, DNA methylation is catalyzed by DNA methylation transferases (DNMT1, DNMT3A, and DNMT3B) and reversed by TET proteins (TET1, TET2, and TET3), which maintain a state of dynamic equilibrium over the course of life (Bhutani et al., 2011).

### Genome-Wide Hypermethylation in AS

Views on genome-wide DNAme state in AS are varied. Hypermethylation may dominate in AS lesions as pointed out in recent reports. With lesion progression, DNAme drifted toward hypermethylation (Valencia-Morales et al., 2015; Lacey et al., 2019)—for example, a recent study finds that DNMT3B-mediated CREG gene hypermethylation becomes a novel mechanism, which may contribute to endothelial dysfunction and atherosclerosis development (Liu et al., 2020).

Silvio Zaina et al. (2014) have examined DNAme levels using whole-genome bisulfite sequencing. They observed that the atherosclerotic portion of the aorta was hypermethylated across many genomic loci in comparison with the matched healthy counterpart. This study using a high-throughput DNA methylation microarray and covering more than 450,000 CpG sites found the repeat element *Alu* to be also hypermethylated. The study also found that TET2 is decreased in atherosclerotic lesion. A deficiency of this study may be its use of post-mortem donor-matched atherosclerotic and non-atherosclerotic portions, which could not better reflect the dynamic changes of arteriosclerosis *in vivo* and do not specifically describe the level of gene methylation in live exact tissues.

Another study (Sharma et al., 2014) collected blood samples from angiographically confirmed CAD patients and healthy controls. It has identified 72 different methylated regions that were hypermethylated in CAD patients in the background of varying homocysteine levels and found methylation to be significantly higher in CAD cases. Though this study took blood from living persons as research objects, a high level of homocysteine could enhance the DNMT expression level, which leads to hypermethylation and affects the judgment results.

### Genome-Wide Hypomethylation in AS

The occurrence of DNA methylation patterns exists in inter-individual variation; genome-wide hypomethylation also exists in AS*.* Wang et al. (2018) collected various tissues from six patients who underwent coronary artery bypass surgery, including atherosclerotic plaques, great saphenous vein, and internal mammary artery. They have found that the genes which participated in immune response-associated pathways (cytokine–cytokine receptor interactions and the MAPK signaling pathway) in AS were enriched by the hypomethylated genes. However, the limitations of this study are the relatively small sample size and the fact that data may be biased because of the possible effects of the drugs given to the patients for treatment. Other studies have found that genes hypomethylated in AS may include keratin gene, ATP-binding proteins, cells of the skeleton, and chromatin regulatory protein-related genes (Aavik et al., 2015).

In conclusion, analyzing multiple cell types involved in atherosclerotic lesions at the single-cell DNAme level for the global methylation status appears to be rather difficult. The DNAme status on gene expression depends on its position in the whole genome, one of the reasons of which is that methylation near transcriptional start sites (TSSs) blocks initiation, but genomic methylation does not block initiation and may even stimulate transcriptional lengthening (Jones, 2012).

Genome-wide methylation in AS only reflected probable methylation levels, but specific “target” genes need to be studied moving forward. More testing tools that have been used, such as NGS, may help to distinguish patterns of DNA methylation of various atherosclerotic tissues and specific epigenetic characteristics (Zhong et al., 2021). (Table 1)

**TABLE 1:** Summary of differences in genome-wide methylation in AS.

Authors	Measurement	DNAme		Reference
Valencia-Morales Mdel P	Microarray	Hypermethylation	2015	Valencia-Morales et al. (2015)
Lacey M	Bisulfite sequencing	Hypermethylation	2019	Lacey et al. (2019)
Liu Y	Bisulfate sequencing	Hypermethylation	2020	Liu et al. (2020)
Zaina *et al*.	Whole-genome bisulfite sequencing	Hypermethylation	2014	Zaina et al. (2014)
Sharma	Bisulfite 454 sequencing	Hypermethylation	2014	Sharma et al. (2014)
Wang X	NGS, microarrays	Hypomethylation	2018	Wang et al. (2018)

### DNA Methylation Link With Lipid Oxidation

AS is a set of processes driven by lipid plaque formation. Hyperlipidemia is considered as the major contributor for the development of atherosclerotic cardiovascular diseases (Zafeiropoulos et al., 2021). Multiple EWASs have explored total cholesterol, low-density lipoprotein cholesterol (LDL-C), and triacylglycerol (TG), which are pro-atherogenic, and high-density lipoprotein cholesterol (HDL-C), which is anti-atherogenic (Hedman et al., 2017). Genes related to lipid metabolism include Kruppel-like factor 2 (KLF2), ATP-binding cassette transporter A1 (ABCA1), and so on.

Lipid oxidation is the initiating factor in the atherogenesis process. The pivotal role of low-density lipoprotein cholesterol (LDL-C) has been well studied and widely recognized. Epigenetic regulation has been observed in low-density lipoprotein oxidation—for example, the upregulated expression of DNMT1 causes methylation of the promoter region of KLF2 in human umbilical vein endothelial cells (HUVECs) treated with oxidized low-density lipoprotein (ox-LDL) (Yan et al., 2017)*.*

The ATP-binding cassette transporter A1 (ABCA1), an important gene associated with lipid metabolism, which could mediate the outflow of phospholipid and cholesterol, binds apolipoprotein A-I (apoA-I) on the cell surface, forms high-density lipoprotein (HDL), reduces the lipid content of plasma membrane free cholesterol, reduces the taxis ability of macrophages, and delays the pathological progress of AS (Ghaznavi et al., 2018). At the same time, ABCA1-mediated cholesterol efflux can change the lipid microenvironment of the cell membrane, activate the anti-inflammatory signaling pathway, and play an important anti-inflammatory role. ABCA1 is a key regulator of cholesterol reverse transport from peripheral tissues back to the liver and participates in the initial stage of cholesterol reverse transport (RCT). Functional defects of ABCA1 may impair the activity of RCT and lead to the formation of foam cells (Annema and Tietge, 2012). In a study of patients with familial hypercholesterolemia, ABCA1 promoter methylation was confirmed to be related to the occurrence of hypercholesterolemia (Peng et al., 2014). In addition, studies have also reported that there is a significant correlation between the methylation level of ABCA1 gene promoter and aging. The abnormally increased methylation level of the ABCA1 gene promoter in elderly patients with stable angina pectoris may be related to the fact that most elderly patients have a long history of atherosclerosis and hyperlipidemia. Prolonged disease states can lead to the accumulation of epigenetic changes (Ghaznavi et al., 2018).

That notwithstanding, the exact molecular mechanisms underlying DNA methylation modulation by lipid oxidation and the association between the global DNA methylation and regulation of atherosclerosis-specific genes are not fully understood. Further investigations to resolve these issues may lead to the identification of novel therapeutic targets to treat atherosclerosis induced by lipid oxidation.

### DNA Methylation Link With Vascular Endothelial Cells in AS

Vascular endothelial cells (ECs) play a key role in local vasodilation, oxygen-free radical generation, and blood vessel homeostasis (Lovren and Verma, 2013). Endothelial dysfunction is one of the main factors causing AS. Studies have found that in all patients, no matter with mild coronary artery disease (CAD) or advanced CAD, endothelium-dependent vasodilation disorder was observed, indicating that endothelial function can be disordered and involved in the entire development of atherosclerosis (Ludmer et al., 1986). Flow shear stress plays an important role in endothelial cell phenotype. In addition, decreased NO bioavailability, vascular oxidative stress, inflammatory response, vascular aging, and hemodynamics can induce endothelial dysfunction and the typical features of early atherosclerotic lesions (Davies et al., 2013).

Unsurprisingly, DNA methylation changes are readily detectable in atherosclerotic tissues—for example, a significant hypomethylation of CpG dinucleotides was measured in the coding region of extracellular superoxide dismutase from rabbit aortic atherosclerotic lesion when compared with normal intima–media (Laukkanen et al., 2002). Proprotein convertase subtilisin/kexin type 9 (PCSK9) plays an important role in cholesterol and fatty acid metabolism. PCSK9 expression in vascular smooth muscle cells (SMCs) and endothelial cells (ECs) reached a maximal value at low shear stress and then began to decline with an increase in shear stress. Researchers found that the PCSK9 promoter DNA methylation may be linked with CAD (Shyamala et al., 2021).

### DNA Methylation and Inflammation in AS

AS is widely known as an inflammatory disease involving immune cell subsets of various lineages, like T cells, B cells, foam cells, and monocytes–macrophages, in which macrophage plays a major role in the progression of lesions (Swirski et al., 2007). During the formation of atherosclerotic plaques, macrophage phagocytosis of oxidized low-density lipoprotein becomes a major contributor to the inflammatory response by secreting pro-inflammatory mediators, eventually dying from necrosis or apoptosis (Davis and Gallagher, 2019). Dying macrophages release their lipid content and tissue factors, then leading to the formation of necrotic prethrombotic cores as a key component of unstable plaques (Davis and Gallagher, 2019).

Macrophages are characterized by a remarkable degree of plasticity, influenced by the affected sites and microenvironment in atherosclerotic lesions, which can be differentiated into M1 and M2 phenotypes through different activation pathways, while the two phenotypes keep a dynamic equilibrium (Jia et al., 2017). M1 macrophage performs a pro-inflammatory character, expressing high levels of pro-inflammatory cytokines such as tumor necrosis factor α, interleukin-6, and interleukin-1β, which predominate in the progression of atherosclerotic lesions and vulnerable plaques (Bakshi et al., 2019). On the contrary, M2-type usually expresses high levels of anti-inflammatory cytokines, such as interleukin-10 and recombinant human arginase-1, plays an anti-inflammatory role, and predominates in stable plaque and early atherosclerosis (Tabas and Bornfeldt, 2016). A significant hallmark of atherosclerosis is the accumulation of pro-inflammatory metabolites in coronary arteries that respond to pro-atherogenic stimuli, such as free fatty acids (FFAs) and oxidized LDLs (ox-LDLs), and the failure to digest lipids that contribute to the formation of foam cells in atherosclerotic plaques (Eshghjoo et al., 2021).

In recent years, growing research has focused on changes in DNA methylation as observed in AS-associated inflammation—for instance, Kumar A and colleagues showed that oxidized LDL (ox-LDL) stimulation of endothelial cells could upregulate DNMT1 and lead to the methylation of the promoter of the gene encoding KLF2. KLF2 plays an important role in vascular endothelial cell immunity and homeostasis. In addition, this effect of ox-LDL on KLF2 methylation can be reversed by treating endothelial cells with the DNMT inhibitor 5-azacytidine (5-azacine) (Kumar et al., 2013). Another important immune regulator, peroxisome proliferator-activated receptor-gamma (PPAR-γ), has anti-inflammatory effects; its dysfunction can lead to lipid accumulation. PPAR-γ is able to promote macrophages to polarize towards M2-like phenotypes and inhibit M1 macrophage polarization. Additional PPAR-γ or pharmacological activation of PPAR-γ effectively prevented DNMT1-induced pro-inflammatory cytokine production in macrophages and AS development in the mouse model. A study in a mouse model found that the DNA methylation status of the proximal PPAR-γ promoter was regulated by DNMT1 in macrophages. The DNMT1-PPARγ pathway in macrophages can regulate chronic inflammation and AS development in mice (Yu et al., 2016).

Similarly, DNA methylation can also affect the development, polarization, and activation of macrophages. It has been observed that the DNA methylation status has changed in monocytes and macrophages during atherogenesis, which may have an influence on monocyte-to-macrophage differentiation and the activation of these two types of cell (Jia et al., 2017). One study compared the gene-specific promoter DNA methylation status of macrophage polarization genes in peripheral blood mononuclear cells (PBMCs) between coronary atherosclerotic heart disease (CHD) cases and controls, showing that the DNA methylation level of M1 macrophage genes was decreased in patients with CAD (Bakshi et al., 2019). This may indicate that the M1 type of pro-inflammatory macrophages may play a dominant role in CHD. Another study involved monocyte chemoattractant protein-1 (MCP-1), which is related to the migration and gathering of monocytes, and found MCP-1 expression due to DNA hypomethylation induced by Hcys mediated by NF-κB/DNMT1 (Wang et al., 2013).

In summary, the methylation of inflammatory cells in AS is mainly manifested as hypomethylation of pro-inflammatory factors and hypermethylation of anti-inflammatory factors, which is particularly consistent with the aggregation of inflammatory cells and adhesion to ECs in the progression of AS.

### Aberrant DNA Methylation in VSMCs

Vascular smooth muscle cell (VSMC) is one of the main cell types in blood vessel wall, and VSMC proliferation and apoptosis are involved in the development of atherosclerosis (Clarke and Bennett, 2006). During the development of AS, VSMCs can convert to multiple phenotypes, including calcification (osteogenesis, chondrogenesis, and osteoclysis) and macrophage surface type. On the one hand, some VSMCs acquire macrophages during phenotypic transformation, characteristically differentiating into lipid-loaded foam cell-like macrophages through healthy low ability to clear lipids, dead cells, and necrotic debris and the exacerbation of inflammation to promote atherosclerosis. On the other hand, VSMCs tend to calcify phenotypic transformation. Phenotypic plasticity and osteochondral differentiation in VSMCs play a key role in atherosclerotic intima calcification (Harith et al., 2016). Intimal calcification and atherosclerotic adverse events, such as plaque rupture, myocardial infarction, stroke and other important relationship. VSMCs regulate phenotypes promotes cell proliferation, which is involved in the whole process of AS (Elia et al., 2019).

Recently, studies have demonstrated that methylation modulated the atherogenic profile also of VSMCs. They handled primary VSMCs with platelet-derived growth factor-BB strongly expressed UHRF1, in cooperation with DNMT1, and found positively modulated the methylation status of different genes associated with VSMC differentiation, such as smooth muscle actin 2, smooth muscle-myosin heavy chain 11 and some proteins (Elia et al., 2018). Strong plasticity and phenotypic transformation of VSMCs play an important role in the formation of AS. Focal adhesion kinase (FAK) could activation elicits VSMC dedifferentiation by stabilizing DNA methyltransferase 3A (DNMT3A). Reduced DNMT3A protein led to DNA hypomethylation in contractile gene promoters, which increased VSMC contractile protein expression. It was also observed that VSMCs in human atherosclerotic lesions show reduced nuclear FAK, which is associated with increased DNMT3A levels and decreased contractile gene expression (Jeong et al., 2021).

To sum up, DNA methylation was observed in nearly all of the pathogenesis of atherosclerosis plaque. The findings of DNA methylation changes may open avenues for novel treatment for CAD management.

### Risk Factors Associated With AS and Methylation

AS is a common clinical disease often associated with other diseases as their end-stage complication—for example, hyperlipidemia, diabetes mellitus, hyperhomocysteinemia, and hyperuricemia (Jiang et al., 2021). In this article, we want to highlight some relevant risk factors that are well known in current studies.

### DNA Methylation Changes Associated With Oxidative Stress

Oxidative stress has been widely accepted as another important risk factor for atherosclerotic cardiovascular diseases (CVD) (Salvayre et al., 2016). Many CVD have been associated, in part or completely, with oxidative stress. Oxidative stress is a process which is damaging to various organs and tissues and caused by reactive oxygen species at the cellular level.

Reactive oxygen species (ROS) accounted for an important proportion. ROS, one important product generated by redox reaction with one or more unpaired electrons in the external shell, includes super oxygen anion $\left(O_{2}^{-}\right)$ and hydrogen peroxide (H_2_O_2_) (Chen et al., 2007). Various metabolic disorders in the body and aging contribute to the imbalance between oxidative and antioxidant mechanisms, leading to ROS-mediated damage (Izzo et al., 2021).

In the cardiovascular system, the excessive formation of $O_{2}^{-}$ leads to the oxidative inactivation of NO and a decrease in its bioavailability (Sazonova et al., 2021). NO has a variety of vascular-protective effects, ranging from vasodilation, anti-aggregation, and anti-inflammatory effects to inhibition of lipid oxidation and vascular smooth muscle hyperplasia (Ritchie et al., 2017). Therefore, the reduction of NO production has a directly promoting effect on atherosclerosis. In addition, oxidative stress plays an important role in lipid peroxidation. Low-density lipoprotein (LDL) peroxidation to oxidized low-density lipoprotein (ox-LDL) accumulates in blood vessels, leading to apoptosis and necrosis of foam cells, forming a necrotic lipid core, generating unstable atherosclerotic plaque, and triggering acute cardiovascular events (Li et al., 2018). Furthermore, studies have found that ox-LDL could increase the formation of ROS in cells in a dose- and time-dependent manner (Chen et al., 2007). In general, oxidative stress promotes the occurrence and development of AS by inhibiting NO production and promoting lipid peroxidation in a pathophysiological perspective.

From a genetic point of view, oxidative stress would cause genetic damage and abnormal DNA demethylation. Due to high oxidation, ROS and its complexes could oxidize large molecules, such as DNA, lipids, and proteins, and alter biological functions. Notably, ROS can induce abnormalities in precise DNA methylation patterns by directly damaging the DNA. One of the most common oxidized derivatives of oxidative damage to DNA is guanine bases from the oxidation product 8-hydroxy-2′-deoxyguanosine (8-OHDG), which is often used as a marker for the determination of oxidative damage (O'Hagan et al., 2011). After DNA damage, methylation patterns were reconstructed during repair, and DNMT1 and related complexes enrichment in chromatin at the damaged site was observed. On the other hand, oxidative damage can also interfere the methylation patterns by inducing abnormalities in DNA demethylation (Niu et al., 2015). The pivotal epigenetic enzyme TET protein catalyzes the demethylation of 5-MC hydroxyethyl to 5-hydroxymethyl cytosine (5-hMC), utilizing Fe(II) as co-factors (Tahiliani et al., 2009), while H_2_O_2_ inhibits the reduction of ferric iron to ferric iron (Niu et al., 2015). As a result, the demethylation of TET protein decreased, and the methylation pattern changed. For atherosclerotic lesions, ROS usually alters DNA methylation markers in a similar manner.

Fortunately, within the blood vessels, there exist antioxidant molecules and systems which become a powerful weapon against oxidative damage. Antioxidants work by reducing ROS production and removing or degrading ROS and/or other oxidants. There are many endogenous and exogenous small-molecule antioxidants like uric acid, glutathione, bilirubin, coenzyme Q, lipoid acid, melatonin, anthocyans, and polyphenols (Salvayre et al., 2016). Another important antioxidant system in the body is superoxide dismutase (Sod) family including Sod1, 2, and 3. Researchers have found that increased oxidative stress in older mice causes hypomethylation in the promoter of Sod2 (encoded mitochondrial Mn-SOD). Aged mice suffer from an altered endothelial function, but NO-dependent dilatory pathway was not observed probably because the Mn-SOD antioxidant enzyme maintains the overall endothelial function (Nguyen et al., 2016).

Briefly speaking, oxidative stress not only inhibits the production of NO and promotes lipid peroxidation but also directly causes oxidative damage to DNA bases and induce the abnormal demethylation of TNT enzyme to disrupt DNA methylation patterns, more or less accelerating the progression of AS.

### Changes in DNA Methylation Associated With Aging

AS is a chronic process, with a progressive course over many years, but it can cause acute clinical events, including acute coronary syndromes (ACS), myocardial infarction (MI), and stroke (Ferreira et al., 2021). Accumulating evidence links cardiovascular aging to epigenetic alterations encompassing a complex interplay of DNA methylation, histone posttranslational modifications, and dynamic nucleosome occupancy governed by numerous epigenetic factors (Zhang et al., 2018). With increasing age, the body is exposed to more and more external stimulus factors such as oxidative stress, smoking (Yang et al., 2019), sitting, and mental stress. Changes in diet and lifestyle contribute to endothelial damage, vasoconstriction, and a series of physiological changes. In this process, epigenetic modification, particularly DNA methylation, occurs, which can well explain the relationship between gene expression and response to the changing environment in adulthood (Cavalli and Heard, 2019). Therefore, understanding the epigenetic mechanism may better explain the aging process (Pagiatakis et al., 2021). Nowadays, DNA methylation is considered to be a significant biomarker of health linked with age (Wang et al., 2020).

Firstly, some common signs have been found in aging individuals, such as genetic material change, including genomic instability increases, telomere shortening, epigenetic change, then nutrition metabolism disorder, mitochondrial dysfunction, aging cells and organelles, and interstitial cell signaling changes. These characteristics in atherosclerosis disease also have a certain embodiment (López-Otín et al., 2013). In this article, we focus on epigenetic changes with aging. Epigenetic drift and epigenetic clock are two phenomena underlying the relationship between DNA methylation and aging (Field et al., 2018). Epigenetic drift means real-time DNA methylation changes are associated with age in an individual but not common across all individuals. The epigenetic clock, on the other hand, represents those sites that are associated with age across all individuals (Jones et al., 2015), so that is one way to understand why patients of the same age may have different levels of methylation. As mentioned earlier, in AS lesions, global methylation level showed an age-dependent decreasing trend, and hypermethylation at some gene sites was observed.

Secondly, vascular senescence is another important feature of body senescence, which is usually manifested by endothelial dysfunction and decreased elasticity of vascular sclerosis (Jin et al., 2019). During vascular aging, DNA methylation also contributes to oxidative stress in the blood vessels. The expression of endogenous nitric oxide synthase (NOS3/eNOS) is specific to ECs in assisting to produce NO and regulate the vascular function (Ambrosini et al., 2020). The promoter region of NOS3 gene encoding eNOS has a low methylation level under physiological state, while hypermethylation occurs under a pathological state, inhibiting the expression of NOS3 and the production of NO.

Thirdly, aging could lead to impaired vascular mitochondrial function, and enhanced mitophagy accelerates AS (Tyrrell et al., 2020). Mitochondrial damage is one of the important markers of aging. Interestingly, recent studies have found that mitochondrial DNA copy number can affect human mortality and cardiovascular disease by changing the nuclear DNA methylation pattern, causing the differential expression of specific genes and changing the signal transduction (Castellani et al., 2020).

Furthermore, aging affects the immune system in many ways, and immune system disorders are crucial in the process of AS lesions. With the increase of age, the regenerative ability of bone marrow hematopoietic stem cells decreases, but the absolute number of various immune cells (such as macrophages, T cells, B cells, *etc*.) increases as their function decreases, leading to a decrease of anti-inflammatory ability (Nikolich-Žugich, 2018). What is more, the number of megakaryocytes increases, which could produce platelets, leading to thrombosis. With the increase of the release of inflammatory mediators *in vivo*, the body is in a state of chronic inflammation for a long time, which will accelerate the occurrence of inflammation-related diseases, such as atherosclerotic diseases. On the other hand, a recent study compared the DNA methylation level of monocytes in young and old people and showed that there was hypomethylation at the CpG sites related to the molecules of human leukocyte antigen (HLA) in the elderly, which may affect the anti-inflammatory ability (Saare et al., 2020).

### DNA Methylation Influenced by High Homocysteine

Homocysteine (HCys) has proved to be associated with cardiovascular disease for decades (Skovierova et al., 2016). Under normal conditions, HCys binds to plasma proteins to form a complex, with less than 1% in the free state (Skovierova et al., 2016). When necessary factors of metabolism like vitamin B_6_, vitamin B_12_, and folic acid are deficient or if methionine intake is excessive, too much HCys is produced and cannot be metabolized out of the body, leading to HCys accumulation (Fiorito et al., 2014). The plasma homocysteine (free HCys) concentrations in normal adults range from 5 to 10 μmol/L and do not exceed 15 μmol/L. Clinically, elevations above 15 μmol/L are defined as hyperhomocysteinemia (HHCys) (Tinelli et al., 2019). HHCys is an independent risk factor for various cardiovascular diseases, particularly vascular endothelial injury leading to AS. Persistent HHCys can increase the production of reactive oxygen species and cause oxidative stress injury and apoptosis of endothelial cells. It can also cause lipid accumulation, inhibit fibrinolysis, and promote thrombosis. In addition, HHCys can also affect the progression of AS disease through epigenetic mechanisms by changing the dynamic balance of SAM/SAH, leading to DNA methylation or hypermethylation.

HCys is closely related to the biosynthesis and metabolism of methionine (Met), which plays an important role in cell life metabolism. The Met cycle produces SAM as a methyl donor and, at the same time, HCys as a by-product. After SAM participates in the methylation reaction, S-adenosine homocysteine (SAH) is generated (Aavik et al., 2019). High levels of SAH in cells will inhibit DNA methylation by binding to DNA methylation enzymes and result in a passive loss of methylation in triplicating DNA. Thus, the dynamic balance of SAM/SAH affects the global methylation level (Shen et al., 2020). The HCys so obtained can be metabolized by two reactions. In re-methylation, HCys can be remethylated to methionine, catalyzed by transmethylase, in most tissues. This process requires folic acid and vitamin B12 as cofactors. Another way is called trans-sulfuration; through this way, cysteine will be produced to continue the metabolism catalyzed by cysteine-β-synthase requiring vitamin B6 as a cofactor in a small number of tissues (Tinelli et al., 2019). In summary, an increased level of HCys would interfere with the methionine cycle, and an increased level of HCys is widely associated with DNA hypomethylation.

As an independent predisposing factor of coronary heart disease, HCys can affect smooth muscle cell proliferation, promote endothelial cell dysfunction, and increase inflammatory mediators (Tinelli et al., 2019). Platelet growth factor (PDGF) can promote the proliferation of vascular endothelial cells. Studies on cultured human umbilical vein endothelial cells (VSMC) *in vitro* showed that increased HCys can induce the hypomethylation of the PDGF promoter, increase mRNA and protein expression levels, and promote the proliferation of VSMC (Han et al., 2014).

Otherwise, as a strong oxidant, HCys can induce the release of ROS, C-reactive protein (CRP), and other superoxide anions through the MAPK, NF-κ B, and other pathways. HCys inhibit the production of nitrogen monoxide (NO), the most powerful vasodilator produced by the endothelium and by the increase of oxidative stress following the production of ROS (Luo et al., 2012). The linkage protein *p66shc* could promote the oxidative stress response of tissue cells and decrease the level of nitric oxide synthase *in vivo*, worsening the apoptosis of endothelial cells. Studies have found that the concentration of HCys is positively correlated with the concentration of *p66shc* mainly because the increase of HCys will lead to the decrease of the methylation level of *p66shc* promoter (Xiao et al., 2019).

In addition, high levels of homocysteine lead to hypermethylation in the promoter region of the estrogen receptor-alpha (ER-а) gene. ER-α are considered human atheroprotective genes regulating the beneficial estrogenic effects on ECs and SMCs (Grimaldi et al., 2015).

While the level of HCys is affected by the level of nutrient supply, such as one-carbon unit donor nutrient folic acid, deficiencies in folic acid and other nutrients, such as vitamins B6 and B12, will increase the homocysteine levels and induce endothelial dysfunction (Hou and Zhao, 2021). Therefore, supplemental nutrients are needed, which can improve the DNA methylation status, reduce the level of inflammatory factors, and delay the progression of atherosclerosis (Hou and Zhao, 2021). A meta-regression analysis reported a positive association between supplementary folic acid dose and methylation levels; folic acid was also the only identified factor among other nutrient supplements (ElGendy et al., 2018). In mice, a folate-deficient diet can influence the mRNA levels of one-carbon metabolic and epigenetic enzymes and reduce the levels of SAM (Bahous et al., 2019). In theory, folic acid deficiency decreased the methyl donors and methylation levels. However, in large human, randomized, controlled clinical trials, it was found that folic acid supplementation reduced the serum homocysteine levels relative to placebo and did not delay the progression of AS (van Dijk et al., 2015). There is no clear evidence that the treatment for hyperhomocysteinemia alters the methylation process and has an exactly curative effect to CHD.

In conclusion, Hcys influenced AS in various ways, such as interfering endothelial cell homeostasis, oxidative stress, and affecting DNA methylation dynamics by SAM/SAH (Table 2).

**TABLE 2:** Atherosclerosis-specific genes modulated *via* DNA methylation during the disease.

	Gene	Involvement	DNAme level of AS	Reference
Lipid oxidation	KLF2	Immune and homeostasis	Hypermethylation	Zafeiropoulos et al. (2021)
	ABCA1	Outflow of phospholipid and cholesterol	Hypermethylation	Hedman et al. (2017)
Endothelial dysfunction	PCSK9	Cholesterol and fatty acid metabolism	Hypermethylation	Davies et al. (2013)
Inflammation (macrophage)	KLF2	Inflammatory response	Hypermethylation	Zafeiropoulos et al. (2021)
	PPAR-γ	Anti-inflammatory	Hypermethylation	Eshghjoo et al. (2021)
	MCP-1	Migration and gathering of monocytes	Hypomethylation	Kumar et al. (2013)
VSMC	Contractile protein	Vasoconstriction	Hypomethylation	Elia et al. (2019)
Oxidative stress	Mn-SOD	Antioxidant	Hypomethylation	O'Hagan et al. (2011)
Aging	NOS3	Produce NO	Hypermethylation	Jones et al. (2015)
	HLA	Anti-inflammatory	Hypomethylation	Castellani et al. (2020)
Hyperhomocysteinemia	PDGF p66shc	Proliferation of VECs	Hypomethylation	Tinelli et al. (2019)
	ER-а	Decrease the level of NO synthase Atheroprotective	Hypomethylation	Han et al. (2014)
			Hypermethylation	Luo et al. (2012)

### Clinical Study and Limitation

In the clinical treatment of atherosclerotic diseases, lowering the blood lipid level is still the most important means for the treatment of related diseases, but pure anti-inflammatory drugs and those with lipid-lowering effects are increasingly limited.

At present, drugs targeting the epigenetic mechanisms have been well developed. Azacytidine, a DNA methyltransferase inhibitor, has been reported to play a certain role in the treatment of hematological tumors (Falchi et al., 2020). When it comes to treatment of cardiovascular diseases, it may play a role in reducing pathological vascular remodeling (Strand et al., 2020). Another potent DNMTs inhibitor, 5-aza-2′-deoxycytidine (5-aza-dC), has been successfully used in the treatment of leukemia and lymphoma. Studies have shown that it can inhibit the macrophages but raise and activate other immune cells to improve AS lesions (Cao et al., 2014), but there have been no reports to determine whether the two drugs can be used in the clinical treatment of AS.

Drug therapy targeting methylation has not been specifically used in AS, possibly because methylation is a widely regulated mode in various tissues, and precision treatment cannot be well achieved. Therefore, more significant further studies of DNA methylation patterns may help to find some new biological diagnostic markers and guide future clinical approaches to treat the disease.

## Conclusion

AS is a major cause of morbidity and mortality all over the world. The basic pathological process involves lipid accumulation together with a maladaptive immune response and alterations of vascular cells within the arterial wall (Jackson et al., 2018).

With modern molecules in the rapid development of biology, genomics, and epigenetics technology, the treatment of diseases is mainly focused on the micro-molecular level, including the study of pathway targets. Nowadays, the regulation of pre-epigenetics has become a new therapeutic target and a hotspot for the AS mechanism—for example, hyperlipidemia can be explained in part by genetics, but not all patients with hyperlipidemia develop an atherosclerotic cardiovascular disease. Some patients with no associated risk factors may still have the disease. Thus, environmental factors (environmental pollution, stress, and insomnia) may epigenetically contribute to the development of atherosclerosis.

As an important supplement to traditional genetics, epigenetics contributes to gene modification through DNA methylation/demethylation, histone modification, and non-coding RNA (Stratton et al., 2019; Yuan et al., 2020). DNA methylation has been studied in depth due to its stable chemical properties and easy detection. Studies have found the expression of abnormal DNA methylation in AS, which plays a role in plaque formation and regulation of monocytes/macrophages. EC and VSMC function and the degree of lesion of AS have important functions. DNA methylation affects the formation of AS by regulating gene expression in some methylated regions and in the differentiation of vascular smooth muscle cells. Research on the methylation of AS-related genes has become a hot trend. The occurrence and development of AS is the result of the combined effects of multiple gene methylation and different methylation levels of different genes.

As observed clinically, the final formation of atherosclerotic lesions is closely related to environmental factors and personal living habits. Epigenetics can well coordinate the relationship between innate genes and acquired environment and influence the disease process from many aspects (Law and Holland, 2019). DNA methylation is an important part of epigenetic modification, and the methylation degree of the gene promoter is inversely correlated with the transcription level. While the “inflammatory immune theory” and “lipid-driven theory” in AS have long been well known, the epigenetic mechanisms are far from understood. Factors like inflammatory stimulation, lipid oxidation, hyperhomocysteinemia, oxidative stress, and aging are all linked to the multi-layered regulation mechanisms of DNA methylation in AS. HHCys and oxidative stress injury are common risk factors for atherosclerotic cardiovascular diseases. HCys, as a by-product of methylation response *in vivo*, can increase or decrease the methylation level by interfering with SAM/SAH dynamic balance. Oxidative stress can induce the formation of new methylation patterns through direct oxidative damage to DNA and interfere with the demethylation process to change the DNA methylation patterns. The aging process is often accompanied by a gradual decline in genome-wide methylation, but the genes involved in atherosclerotic protection show abnormal hypermethylation.

Other epigenetic regulatory mechanisms were not discussed, such as ATP-dependent chromatin-remodeling complexes, DNA and histone modifications, and non-coding RNAs, which also play an important role in the etiology and pathogenesis of AS.

## References

[B1] Aavik E. Babu M. Ylä-Herttuala S. (2019). DNA Methylation Processes in Atheosclerotic Plaque. Atherosclerosis 281, 168–179. 10.1016/j.atherosclerosis.2018.12.006 30591183

[B2] Aavik E. Lumivuori H. Leppanen O. Wirth T. Hakkinen S.-K. Brasen J.-H. (2015). Global DNA Methylation Analysis of Human Atherosclerotic Plaques Reveals Extensive Genomic Hypomethylation and Reactivation at Imprinted Locus 14q32 Involving Induction of a miRNA Cluster. %J Eur. Heart J. 36, 993–1000. 10.1093/eurheartj/ehu437 25411193

[B3] Ambrosini S. Mohammed S. A. Lüscher T. F. Costantino S. Paneni F. (2020). New Mechanisms of Vascular Dysfunction in Cardiometabolic Patients: Focus on Epigenetics. High Blood Press. Cardiovasc. Prev. 27, 363–371. 10.1007/s40292-020-00400-2 32740853

[B4] Annema W. Tietge U. J. (2012). Regulation of Reverse Cholesterol Transport - a Comprehensive Appraisal of Available Animal Studies. Nutr. Metab. 9, 25. 10.1186/1743-7075-9-25 PMC3366910 22458435

[B5] Bahous R. H. Cosín-Tomás M. Deng L. Leclerc D. Malysheva O. Ho M. K. (2019). Early Manifestations of Brain Aging in Mice Due to Low Dietary Folate and Mild MTHFR Deficiency. Mol. Neurobiol. 56, 4175–4191. 10.1007/s12035-018-1375-3 30288696

[B6] Bakshi C. Vijayvergiya R. Dhawan V. (2019). Aberrant DNA Methylation of M1-Macrophage Genes in Coronary Artery Disease. Sci. Rep. 9, 1429. 10.1038/s41598-018-38040-1 30723273 PMC6363807

[B7] Bhutani N. Burns D. M. Blau H. M. (2011). DNA Demethylation Dynamics. Cell 146, 866–872. 10.1016/j.cell.2011.08.042 21925312 PMC3236603

[B8] Botts S. R. Fish J. E. Howe K. L. (2021). Dysfunctional Vascular Endothelium as a Driver of Atherosclerosis: Emerging Insights into Pathogenesis and Treatment. Front. Pharmacol. 12, 787541. 10.3389/fphar.2021.787541 35002720 PMC8727904

[B9] Cao Q. Wang X. Jia L. Mondal A. K. Diallo A. Hawkins G. A. (2014). Inhibiting DNA Methylation by 5-Aza-2'-Deoxycytidine Ameliorates Atherosclerosis through Suppressing Macrophage Inflammation. Endocrinology 155, 4925–4938. 10.1210/en.2014-1595 25251587 PMC4239421

[B10] Castellani C. A. Longchamps R. J. Sumpter J. A. Newcomb C. E. Lane J. A. Grove M. L. (2020). Mitochondrial DNA Copy Number Can Influence Mortality and Cardiovascular Disease via Methylation of Nuclear DNA CpGs. Genome Med. 12, 84. 10.1186/s13073-020-00778-7 32988399 PMC7523322

[B11] Cavalli G. Heard E. (2019). Advances in Epigenetics Link Genetics to the Environment and Disease. Nature 571, 489–499. 10.1038/s41586-019-1411-0 31341302

[B12] Chen T. Ueda Y. Dodge J. E. Wang Z. Li E. (2003). Establishment and Maintenance of Genomic Methylation Patterns in Mouse Embryonic Stem Cells by Dnmt3a and Dnmt3b. Mol. Cel Biol 23, 5594–5605. 10.1128/mcb.23.16.5594-5605.2003 PMC166327 12897133

[B13] Chen X. P. Xun K. L. Wu Q. Zhang T. T. Shi J. S. Du G. H. (2007). Oxidized Low Density Lipoprotein Receptor-1 Mediates Oxidized Low Density Lipoprotein-Induced Apoptosis in Human Umbilical Vein Endothelial Cells: Role of Reactive Oxygen Species. Vascul Pharmacol. 47, 1–9. 10.1016/j.vph.2007.01.004 17433786

[B14] Chistiakov D. A. Orekhov A. N. Bobryshev Y. V. (2017). Treatment of Cardiovascular Pathology with Epigenetically Active Agents: Focus on Natural and Synthetic Inhibitors of DNA Methylation and Histone Deacetylation. Int. J. Cardiol. 227, 66–82. 10.1016/j.ijcard.2016.11.204 27852009

[B15] Clarke M. Bennett M. (2006). The Emerging Role of Vascular Smooth Muscle Cell Apoptosis in Atherosclerosis and Plaque Stability. Am. J. Nephrol. 26, 531–535. 10.1159/000097815 17159340

[B16] Davies P. F. Civelek M. Fang Y. Fleming I. (2013). The Atherosusceptible Endothelium: Endothelial Phenotypes in Complex Haemodynamic Shear Stress Regions In Vivo. Cardiovasc. Res. 99, 315–327. 10.1093/cvr/cvt101 23619421 PMC3695748

[B17] Davis F. M. Gallagher K. A. (2019). Epigenetic Mechanisms in Monocytes/Macrophages Regulate Inflammation in Cardiometabolic and Vascular Disease. Arterioscler Thromb. Vasc. Biol. 39, 623–634. 10.1161/ATVBAHA.118.312135 30760015 PMC6438376

[B18] Ehrlich M. (2019). DNA Hypermethylation in Disease: Mechanisms and Clinical Relevance. Epigenetics 14, 1141–1163. 10.1080/15592294.2019.1638701 31284823 PMC6791695

[B19] ElGendy K. Malcomson F. C. Lara J. G. Bradburn D. M. Mathers J. C. (2018). Effects of Dietary Interventions on DNA Methylation in Adult Humans: Systematic Review and Meta-Analysis. Br. J. Nutr. 120, 961–976. 10.1017/S000711451800243X 30355391

[B20] Elia L. Kunderfranco P. Carullo P. Vacchiano M. Farina F. M. Hall I. F. (2018). UHRF1 Epigenetically Orchestrates Smooth Muscle Cell Plasticity in Arterial Disease. J. Clin. Invest. 128, 2473–2486. 10.1172/JCI96121 29558369 PMC5983314

[B21] Elia L. Condorelli G. Biology C. (2019). The Involvement of Epigenetics in Vascular Disease Development. Int. J. Biochem. Cel Biol 107, 27–31. 10.1016/j.biocel.2018.12.005 30543933

[B22] Eshghjoo S. Jayaraman A. Sun Y. Alaniz R. C. (2021). Microbiota-Mediated Immune Regulation in Atherosclerosis. Molecules 26. 10.3390/molecules26010179 PMC7795654 33401401

[B23] Falchi L. Ma H. Klein S. Lue J. K. Montanari F. Marchi E. (2020). Combined Oral 5-Azacytidine and Romidepsin Are Highly Effective in Patients with PTCL: A Multicenter Phase 2 Study. Blood 137, 2161–2170. 10.1182/blood.2020009004 33171487

[B24] Ferreira J. P. Cleland J. G. Lam C. S. P. Anker S. D. Mehra M. R. van Veldhuisen D. J. (2021). Heart Failure Re-hospitalizations and Subsequent Fatal Events in Coronary Artery Disease: Insights from COMMANDER-HF, EPHESUS, and EXAMINE. Clin. Res. Cardiol. 110, 1554–1563. 10.1007/s00392-021-01830-1 33686472

[B25] Field A. E. Robertson N. A. Wang T. Havas A. Ideker T. Adams P. D. (2018). DNA Methylation Clocks in Aging: Categories, Causes, and Consequences. Mol. Cel 71, 882–895. 10.1016/j.molcel.2018.08.008 PMC6520108 30241605

[B26] Fiorito G. Guarrera S. Valle C. Ricceri F. Russo A. Grioni S. (2014). B-vitamins Intake, DNA-Methylation of One Carbon Metabolism and Homocysteine Pathway Genes and Myocardial Infarction Risk: the EPICOR Study. Nutr. Metab. Cardiovasc. Dis. 24, 483–488. 10.1016/j.numecd.2013.10.026 24418380

[B27] Ghaznavi H. Mahmoodi K. Soltanpour M. S. (2018). A Preliminary Study of the Association between the ABCA1 Gene Promoter DNA Methylation and Coronary Artery Disease Risk. Mol. Biol. Res. Commun. 7, 59–65. 10.22099/mbrc.2018.28910.1312 30046619 PMC6054780

[B28] Grimaldi V. Vietri M. T. Schiano C. Picascia A. De Pascale M. R. Fiorito C. (2015). Epigenetic Reprogramming in Atherosclerosis. Curr. Atheroscler. Rep. 17, 476. 10.1007/s11883-014-0476-3 25433555

[B29] Gujar H. Weisenberger D. J. Liang G. (2019). The Roles of Human DNA Methyltransferases and Their Isoforms in Shaping the Epigenome. Genes (Basel) 10, 172. 10.3390/genes10020172 PMC6409524 30813436

[B30] Han X. B. Zhang H. P. Cao C. J. Wang Y. H. Tian J. Yang X. L. (2014). Aberrant DNA Methylation of the PDGF Gene in Homocysteine-Mediated VSMC Proliferation and its Underlying Mechanism. Mol. Med. Rep. 10, 947–954. 10.3892/mmr.2014.2249 24841643

[B31] Harith H. H. Di Bartolo B. A. Cartland S. P. Genner S. Kavurma M. M. (2016). Insulin Promotes Vascular Smooth Muscle Cell Proliferation and Apoptosis via Differential Regulation of Tumor Necrosis Factor-Related Apoptosis-Inducing Ligand. J. Diabetes 8, 568–578. 10.1111/1753-0407.12339 26333348

[B32] Hedman Å Mendelson M. Marioni R. Gustafsson S. Joehanes R. Irvin M. R. (2017). Epigenetic Patterns in Blood Associated with Lipid Traits Predict Incident Coronary Heart Disease Events and Are Enriched for Results from Genome-wide Association Studies. Circ. Cardiovasc. Genet. 10, e001487. 10.1161/circgenetics.116.001487 28213390 PMC5331877

[B33] Hou H. Zhao H. (2021). Epigenetic Factors in Atherosclerosis: DNA Methylation, Folic Acid Metabolism, and Intestinal Microbiota. Clin. Chim. Acta 512, 7–11. 10.1016/j.cca.2020.11.013 33232735

[B34] Izzo C. Vitillo P. Di Pietro P. Visco V. Strianese A. Virtuoso N. (2021). The Role of Oxidative Stress in Cardiovascular Aging and Cardiovascular Diseases. Life (Basel) 11, 60. 10.3390/life11010060 33467601 PMC7829951

[B35] Jackson A. O. Regine M. A. Subrata C. Long S. (2018). Molecular Mechanisms and Genetic Regulation in Atherosclerosis. Int. J. Cardiol. Heart Vasc. 21, 36–44. 10.1016/j.ijcha.2018.09.006 30276232 PMC6161413

[B36] Jeong K. Murphy J. Kim J. Campbell P. M. Park H. Rodriguez Y. A. R. (2021). FAK Activation Promotes SMC Dedifferentiation via Increased DNA Methylation in Contractile Genes. Circ. Res. 129, e215–e233. 10.1161/circresaha.121.319066 34702049 PMC8639767

[B37] Jia S. J. Gao K. Q. Zhao M. (2017). Epigenetic Regulation in Monocyte/macrophage: A Key Player during Atherosclerosis. Cardiovasc. Ther. 35, e12262. 10.1111/1755-5922.12262 28371472

[B38] Jiang W. Chen M. Huang J. Shang Y. Qin C. Ruan Z. (2021). Proteinuria Is Independently Associated with Carotid Atherosclerosis: a Multicentric Study. BMC Cardiovasc. Disord. 21, 554. 10.1186/s12872-021-02367-x 34798829 PMC8603343

[B39] Jin J. Liu Y. Huang L. Tan H. (2019). Advances in Epigenetic Regulation of Vascular Aging. Rev. Cardiovasc. Med. 20, 19–25. 10.31083/j.rcm.2019.01.3189 31184092

[B40] Jones M. J. Goodman S. J. Kobor M. S. (2015). DNA Methylation and Healthy Human Aging. Aging Cell 14, 924–932. 10.1111/acel.12349 25913071 PMC4693469

[B41] Jones P. A. (2012). Functions of DNA Methylation: Islands, Start Sites, Gene Bodies and beyond. Nat. Rev. Genet. 13, 484–492. 10.1038/nrg3230 22641018

[B42] Khyzha N. Alizada A. Wilson M. D. Fish J. E. (2017). Epigenetics of Atherosclerosis: Emerging Mechanisms and Methods. Trends Mol. Med. 23, 332–347. 10.1016/j.molmed.2017.02.004 28291707

[B43] Kumar A. Kumar S. Vikram A. Hoffman T. A. Naqvi A. Lewarchik C. M. (2013). Histone and DNA Methylation-Mediated Epigenetic Downregulation of Endothelial Kruppel-like Factor 2 by Low-Density Lipoprotein Cholesterol. Arterioscler Thromb. Vasc. Biol. 33, 1936–1942. 10.1161/ATVBAHA.113.301765 23723375

[B44] Lacey M. Baribault C. Ehrlich K. C. Ehrlich M. (2019). Atherosclerosis-associated Differentially Methylated Regions Can Reflect the Disease Phenotype and Are Often at Enhancers. Atherosclerosis 280, 183–191. 10.1016/j.atherosclerosis.2018.11.031 30529831 PMC6348116

[B45] Laukkanen M. O. Kivelä A. Rissanen T. Rutanen J. Karkkainen M. K. Leppanen O. (2002). Adenovirus-mediated Extracellular Superoxide Dismutase Gene Therapy Reduces Neointima Formation in Balloon-Denuded Rabbit Aorta. Circulation 106, 1999–2003. 10.1161/01.cir.0000031331.05368.9d 12370226

[B46] Law P. P. Holland M. L. (2019). DNA Methylation at the Crossroads of Gene and Environment Interactions. Essays Biochem. 63, 717–726. 10.1042/EBC20190031 31782496 PMC6923319

[B47] Li D. Yan J. Yuan Y. Wang C. Wu J. Chen Q. (2018). Genome-wide DNA Methylome Alterations in Acute Coronary Syndrome. Int. J. Mol. Med. 41, 220–232. 10.3892/ijmm.2017.3220 29115576 PMC5746328

[B48] Liu Y. Peng W. Qu K. Lin X. Zeng Z. Chen J. (2018). TET2: A Novel Epigenetic Regulator and Potential Intervention Target for Atherosclerosis. DNA Cel Biol 37, 517–523. 10.1089/dna.2017.4118 PMC5985959 29653065

[B49] Liu Y. Tian X. Liu S. Liu D. Li Y. Liu M. (2020). DNA Hypermethylation: A Novel Mechanism of CREG Gene Suppression and Atherosclerogenic Endothelial Dysfunction. Redox Biol. 32, 101444. 10.1016/j.redox.2020.101444 32067910 PMC7264464

[B50] López-Otín C. Blasco M. A. Partridge L. Serrano M. Kroemer G. (2013). The Hallmarks of Aging. Cell 153, 1194–1217. 10.1016/j.cell.2013.05.039 23746838 PMC3836174

[B51] Lovren F. Verma S. (2013). Evolving Role of Microparticles in the Pathophysiology of Endothelial Dysfunction. Clin. Chem. 59, 1166–1174. 10.1373/clinchem.2012.199711 23529703

[B52] Ludmer P. L. Selwyn A. P. Shook T. L. Wayne R. R. Mudge G. H. Alexander R. W. (1986). Paradoxical Vasoconstriction Induced by Acetylcholine in Atherosclerotic Coronary Arteries. N. Engl. J. Med. 315, 1046–1051. 10.1056/NEJM198610233151702 3093861

[B53] Luo X. Xiao Y. Song F. Yang Y. Xia M. Ling W. (2012). Increased Plasma S-Adenosyl-Homocysteine Levels Induce the Proliferation and Migration of VSMCs through an Oxidative Stress-Erk1/2 Pathway in apoE(-/-) Mice. Cardiovasc. Res. 95, 241–250. 10.1093/cvr/cvs130 22492673

[B54] Nguyen A. Leblond F. Mamarbachi M. Geoffroy S. Thorin E. (2016). Age-Dependent Demethylation of Sod2 Promoter in the Mouse Femoral Artery. Oxid Med. Cel Longev 2016, 8627384. 10.1155/2016/8627384 PMC4771915 26989455

[B55] Nikolich-Žugich J. (2018). The Twilight of Immunity: Emerging Concepts in Aging of the Immune System. J. Nat. Immunol. 19, 10–19. 10.1038/s41590-017-0006-x 29242543

[B56] Niu Y. DesMarais T. L. Tong Z. Yao Y. Costa M. (2015). Oxidative Stress Alters Global Histone Modification and DNA Methylation. Free Radic. Biol. Med. 82, 22–28. 10.1016/j.freeradbiomed.2015.01.028 25656994 PMC4464695

[B57] O'Hagan H. M. Wang W. Sen S. Destefano Shields C. Lee S. S. Zhang Y. W. (2011). Oxidative Damage Targets Complexes Containing DNA Methyltransferases, SIRT1, and Polycomb Members to Promoter CpG Islands. Cancer Cell 20, 606–619. 10.1016/j.ccr.2011.09.012 22094255 PMC3220885

[B58] Pagiatakis C. Musolino E. Gornati R. Bernardini G. Papait R. (2021). Epigenetics of Aging and Disease: a Brief Overview. Aging Clin. Exp. Res. 33, 737–745. 10.1007/s40520-019-01430-0 31811572 PMC8084772

[B59] Papin C. Le Gras S. Ibrahim A. Salem H. Karimi M. M. Stoll I. (2020). CpG Islands Shape the Epigenome Landscape. J. Mol. Biol., 433, 166659. 10.1016/j.jmb.2020.09.018 33010306

[B60] Parry A. Rulands S. Reik W. (2021). Active Turnover of DNA Methylation during Cell Fate Decisions. Nat. Rev. Genet. 22, 59–66. 10.1038/s41576-020-00287-8 33024290

[B61] Patani H. Rushton M. D. Higham J. Teijeiro S. A. Oxley D. Cutillas P. (2020). Transition to Naïve Human Pluripotency Mirrors Pan-Cancer DNA Hypermethylation. Nat. Commun. 11, 3671. 10.1038/s41467-020-17269-3 32699299 PMC7376100

[B62] Peng P. Wang L. Yang X. Huang X. Ba Y. Chen X. (2014). A Preliminary Study of the Relationship between Promoter Methylation of the ABCG1, GALNT2 and HMGCR Genes and Coronary Heart Disease. PLoS One 9, e102265. 10.1371/journal.pone.0102265 25084356 PMC4118847

[B63] Rask-Andersen M. Martinsson D. Ahsan M. Enroth S. Ek W. E. Gyllensten U. (2016). Epigenome-wide Association Study Reveals Differential DNA Methylation in Individuals with a History of Myocardial Infarction. Hum. Mol. Genet. 25, 4739–4748. 10.1093/hmg/ddw302 28172975

[B64] Ritchie R. H. Drummond G. R. Sobey C. G. De Silva T. M. Kemp-Harper B. K. (2017). The Opposing Roles of NO and Oxidative Stress in Cardiovascular Disease. Pharmacol. Res. 116, 57–69. 10.1016/j.phrs.2016.12.017 27988384

[B65] Rizzacasa B. Amati F. Romeo F. Novelli G. Mehta J. L. (2019). Epigenetic Modification in Coronary Atherosclerosis: JACC Review Topic of the Week. J. Am. Coll. Cardiol. 74, 1352–1365. 10.1016/j.jacc.2019.07.043 31488273

[B66] Saare M. Tserel L. Haljasmägi L. Taalberg E. Peet N. Eimre M. (2020). Monocytes Present Age-Related Changes in Phospholipid Concentration and Decreased Energy Metabolism. Aging Cell 19, e13127. 10.1111/acel.13127 32107839 PMC7189998

[B67] Salvayre R. Negre-Salvayre A. Camaré C. (2016). Oxidative Theory of Atherosclerosis and Antioxidants. Biochimie 125, 281–296. 10.1016/j.biochi.2015.12.014 26717905

[B68] Sazonova M. A. Sinyov V. V. Ryzhkova A. I. Sazonova M. D. Kirichenko T. V. Khotina V. A. (2021). Some Molecular and Cellular Stress Mechanisms Associated with Neurodegenerative Diseases and Atherosclerosis. Int. J. Mol. Sci. 22, 699. 10.3390/ijms22020699 PMC7828120 33445687

[B69] Schiano C. Benincasa G. Franzese M. Della Mura N. Pane K. Salvatore M. (2020). Epigenetic-sensitive Pathways in Personalized Therapy of Major Cardiovascular Diseases. Pharmacol. Ther. 210, 107514. 10.1016/j.pharmthera.2020.107514 32105674

[B70] Sharma P. Garg G. Kumar A. Mohammad F. Kumar S. R. Tanwar V. S. (2014). Genome Wide DNA Methylation Profiling for Epigenetic Alteration in Coronary Artery Disease Patients. Gene 541, 31–40. 10.1016/j.gene.2014.02.034 24582973

[B71] Shen W. Gao C. Cueto R. Liu L. Fu H. Shao Y. (2020). Homocysteine-methionine Cycle Is a Metabolic Sensor System Controlling Methylation-Regulated Pathological Signaling. Redox Biol. 28, 101322. 10.1016/j.redox.2019.101322 31605963 PMC6812029

[B72] Shyamala N. Gundapaneni K. Galimudi R. Tupurani M. A. Padala C. Puranam K. (2021). PCSK9 Genetic (Rs11591147) and Epigenetic (DNA Methylation) Modifications Associated with PCSK9 Expression and Serum Proteins in CAD Patients. J. Gene Med. 23, e3346. 10.1002/jgm.3346 33885177

[B73] Skovierova H. Vidomanova E. Mahmood S. Sopková J. Drgová A. Červeňová T. (2016). The Molecular and Cellular Effect of Homocysteine Metabolism Imbalance on Human Health. Int. J. Mol. Sci. 17, 1733. 10.3390/ijms17101733 PMC5085763 27775595

[B74] Strand K. A. Lu S. Mutryn M. F. Li L. Zhou Q. Enyart B. T. (2020). High Throughput Screen Identifies the DNMT1 (DNA Methyltransferase-1) Inhibitor, 5-Azacytidine, as a Potent Inducer of PTEN (Phosphatase and Tensin Homolog): Central Role for PTEN in 5-Azacytidine Protection against Pathological Vascular Remodeling. Arterioscler Thromb. Vasc. Biol. 40, 1854–1869. 10.1161/ATVBAHA.120.314458 32580634 PMC7377968

[B75] Stratton M. S. Farina F. M. Elia L. (2019). Epigenetics and Vascular Diseases. J. Mol. Cel Cardiol 133, 148–163. 10.1016/j.yjmcc.2019.06.010 PMC6757326 31211956

[B76] Swirski F. K. Libby P. Aikawa E. Alcaide P. Luscinskas F. W. Weissleder R. (2007). Ly-6Chi Monocytes Dominate Hypercholesterolemia-Associated Monocytosis and Give Rise to Macrophages in Atheromata. J. Clin. Invest. 117, 195–205. 10.1172/JCI29950 17200719 PMC1716211

[B77] Tabaei S. Tabaee S. S. (2019). DNA Methylation Abnormalities in Atherosclerosis. Artif. Cell Nanomed Biotechnol 47, 2031–2041. 10.1080/21691401.2019.1617724 31116040

[B78] Tabas I. Bornfeldt K. E. (2016). Macrophage Phenotype and Function in Different Stages of Atherosclerosis. Circ. Res. 118, 653–667. 10.1161/CIRCRESAHA.115.306256 26892964 PMC4762068

[B79] Tabas I. García-Cardeña G. Owens G. K. (2015). Recent Insights into the Cellular Biology of Atherosclerosis. J. Cel Biol 209, 13–22. 10.1083/jcb.201412052 PMC4395483 25869663

[B80] Tahiliani M. Koh K. P. Shen Y. Pastor W. A. Bandukwala H. Brudno Y. (2009). Conversion of 5-methylcytosine to 5-hydroxymethylcytosine in Mammalian DNA by MLL Partner TET1. Science 324, 930–935. 10.1126/science.1170116 19372391 PMC2715015

[B81] Tinelli C. Di Pino A. Ficulle E. Marcelli S. Feligioni M. (2019). Hyperhomocysteinemia as a Risk Factor and Potential Nutraceutical Target for Certain Pathologies. Front. Nutr. 6, 49. 10.3389/fnut.2019.00049 31069230 PMC6491750

[B82] Tyrrell D. J. Blin M. G. Song J. Wood S. C. Zhang M. Beard D. A. (2020). Age-Associated Mitochondrial Dysfunction Accelerates Atherogenesis. Circ. Res. 126, 298–314. 10.1161/CIRCRESAHA.119.315644 31818196 PMC7006722

[B83] Valencia-Morales M. P. Zaina S. Heyn H. Carmona F. J. Varol N. Sayols S. (2015). The DNA Methylation Drift of the Atherosclerotic Aorta Increases with Lesion Progression. BMC Med. Genomics 8, 7. 10.1186/s12920-015-0085-1 25881171 PMC4353677

[B84] van Dijk S. C. Enneman A. W. Swart K. M. van Wijngaarden J. P. Ham A. C. Brouwer-Brolsma E. M. (2015). Effects of 2-year Vitamin B12 and Folic Acid Supplementation in Hyperhomocysteinemic Elderly on Arterial Stiffness and Cardiovascular Outcomes within the B-PROOF Trial. J. Hypertens. 33, 1897–1906. discussion 906. 10.1097/HJH.0000000000000647 26147383

[B85] Veland N. Lu Y. Hardikar S. Gaddis S. Zeng Y. Liu B. (2019). DNMT3L Facilitates DNA Methylation Partly by Maintaining DNMT3A Stability in Mouse Embryonic Stem Cells. Nucleic Acids Res. 47, 152–167. 10.1093/nar/gky947 30321403 PMC6326784

[B86] Veljkovic N. Zaric B. Djuric I. Obradovic M. Sudar-Milovanivic E. Radak D. (2018). Genetic Markers for Coronary Artery Disease. Medicina (Kaunas) 54, 36. 10.3390/medicina54030036 PMC6122104 30344267

[B87] Wang J. Jiang Y. Yang A. Sun W. Ma C. Ma S. (2013). Hyperhomocysteinemia-Induced Monocyte Chemoattractant Protein-1 Promoter DNA Methylation by Nuclear Factor-Κb/DNA Methyltransferase 1 in Apolipoprotein E-Deficient Mice. Biores Open Access 2, 118–127. 10.1089/biores.2012.0300 23593564 PMC3620495

[B88] Wang X. Liu A. H. Jia Z. W. Pu K. Chen K. Y. Guo H. (2018). Genome-wide DNA Methylation Patterns in Coronary Heart Disease. Herz 43, 656–662. 10.1007/s00059-017-4616-8 28884387

[B89] Wang C. Ni W. Yao Y. Just A. Heiss J. Wei Y. (2020). DNA Methylation-Based Biomarkers of Age Acceleration and All-Cause Death, Myocardial Infarction, Stroke, and Cancer in Two Cohorts: The NAS, and KORA F4. EBioMedicine 63, 103151. 10.1016/j.ebiom.2020.103151 33279859 PMC7724153

[B90] Xia Y. Brewer A. Bell J. T. (2021). DNA Methylation Signatures of Incident Coronary Heart Disease: Findings from Epigenome-wide Association Studies. Clin. Epigenetics 13, 186. 10.1186/s13148-021-01175-6 34627379 PMC8501606

[B91] Xiao Y. Xia J. Cheng J. Huang H. Zhou Y. Yang X. (2019). Inhibition of S-Adenosylhomocysteine Hydrolase Induces Endothelial Dysfunction via Epigenetic Regulation of P66shc-Mediated Oxidative Stress Pathway. Circulation 139, 2260–2277. 10.1161/CIRCULATIONAHA.118.036336 30773021

[B92] Yan Z. Deng Y. Jiao F. Guo J. Ou H. (2017). Lipopolysaccharide Downregulates Kruppel-like Factor 2 (KLF2) via Inducing DNMT1-Mediated Hypermethylation in Endothelial Cells. Inflammation 40, 1589–1598. 10.1007/s10753-017-0599-0 28578476

[B93] Yang Y. Gao X. Just A. C. Colicino E. Wang C. Coull B. A. (2019). Smoking-Related DNA Methylation Is Associated with DNA Methylation Phenotypic Age Acceleration: The Veterans Affairs Normative Aging Study. Int. J. Environ. Res. Public Health 16, 2356. 10.3390/ijerph16132356 PMC6651499 31277270

[B94] Yu J. Qiu Y. Yang J. Bian S. Chen G. Deng M. (2016). DNMT1-PPARγ Pathway in Macrophages Regulates Chronic Inflammation and Atherosclerosis Development in Mice. Sci. Rep. 6, 30053. 10.1038/srep30053 27530451 PMC4987643

[B95] Yuan Y. Xu L. Geng Z. Liu J. Zhang L. Wu Y. (2020). The Role of Non-coding RNA Network in Atherosclerosis. Life Sci. 265, 118756. 10.1016/j.lfs.2020.118756 33189816

[B96] Zafeiropoulos S. Farmakis I. Kartas A. Arvanitaki A. Pagiantza A. Boulmpou A. (2021). Reinforcing Adherence to Lipid-Lowering Therapy after an Acute Coronary Syndrome: A Pragmatic Randomized Controlled Trial. Atherosclerosis 323, 37–43. 10.1016/j.atherosclerosis.2021.03.013 33780749

[B97] Zaina S. Heyn H. Carmona F. J. Varol N. Sayols S. Condom E. (2014). DNA Methylation Map of Human Atherosclerosis. Circ. Cardiovasc. Genet. 7, 692–700. 10.1161/CIRCGENETICS.113.000441 25091541

[B98] Zhang W. Song M. Qu J. Liu G. H. (2018). Epigenetic Modifications in Cardiovascular Aging and Diseases. Circ. Res. 123, 773–786. 10.1161/CIRCRESAHA.118.312497 30355081

[B99] Zhang Y. Mei J. Li J. Zhang Y. Zhou Q. Xu F. (2021). DNA Methylation in Atherosclerosis: A New Perspective. Evid. Based Complement. Alternat Med. 2021, 6623657. 10.1155/2021/6623657 34257689 PMC8249120

[B100] Zhao D. Liu J. Wang M. Zhang X. Zhou M. (2019). Epidemiology of Cardiovascular Disease in China: Current Features and Implications. Nat. Rev. Cardiol. 16, 203–212. 10.1038/s41569-018-0119-4 30467329

[B101] Zhong Y. Chen L. Li J. Yao Y. Liu Q. Niu K. (2021). Integration of Summary Data from GWAS and eQTL Studies Identified Novel Risk Genes for Coronary Artery Disease. Medicine (Baltimore) 100, e24769. 10.1097/md.0000000000024769 33725943 PMC7982177

